# Genetic variation in Glutathione S-Transferase Omega-1, Arsenic Methyltransferase and Methylene-tetrahydrofolate Reductase, arsenic exposure and bladder cancer: a case–control study

**DOI:** 10.1186/1476-069X-11-43

**Published:** 2012-06-29

**Authors:** Jennifer L Beebe-Dimmer, Priyanka T Iyer, Jerome O Nriagu, Greg R Keele, Shilpin Mehta, Jaymie R Meliker, Ethan M Lange, Ann G Schwartz, Kimberly A Zuhlke, David Schottenfeld, Kathleen A Cooney

**Affiliations:** 1 Program of Population Studies and Disparities Research, Karmanos Cancer Institute, 4100 John R, Detroit, MI 48201, USA; 2 Department of Oncology, Wayne State University, Detroit, MI, USA; 3 Department of Environmental Health Sciences and Epidemiology, University of Michigan, Ann Arbor, MI, USA; 4 Department of Preventive Medicine and Graduate Program in Public Health, StonyBrook University Medical Center, New York, Stony Brook, USA; 5 Department of Genetics, University of North Carolina, Chapel Hill, NC, USA; 6 Department of Biostatistics, University of North Carolina, Chapel Hill, NC, USA; 7 Department of Internal Medicine, University of Michigan Medical School Ann Arbor, Ann Arbor, MI, USA; 8 Department of Epidemiology, University of Michigan, Ann Arbor, MI, USA; 9 Department of Urology, University of Michigan Medical School, Ann Arbor, MI, USA

**Keywords:** Genetic epidemiology, Single nucleotide polymorphisms, Urothelial cancer, Arsenic methylation

## Abstract

**Background:**

Ingestion of groundwater with high concentrations of inorganic arsenic has been linked to adverse health outcomes, including bladder cancer, however studies have not consistently observed any elevation in risk at lower concentrations. Genetic variability in the metabolism and clearance of arsenic is an important consideration in any investigation of its potential health risks. Therefore, we examined the association between genes thought to play a role in the metabolism of arsenic and bladder cancer.

**Methods:**

Single nucleotide polymorphisms (SNPs) in GSTO-1, As3MT and MTHFR were genotyped using DNA from 219 bladder cancer cases and 273 controls participating in a case–control study in Southeastern Michigan and exposed to low to moderate (<50 μg/L) levels of arsenic in their drinking water. A time-weighted measure of arsenic exposure was constructed using measures from household water samples combined with past residential history, geocoded and merged with archived arsenic data predicted from multiple resources.

**Results:**

While no single SNP in As3MT was significantly associated with bladder cancer overall, several SNPs were associated with bladder cancer among those exposed to higher arsenic levels. Individuals with one or more copies of the C allele in rs11191439 (the Met287Thr polymorphism) had an elevated risk of bladder cancer (OR = 1.17; 95% CI = 1.04-1.32 per 1 μg/L increase in average exposure). However, no association was observed between average arsenic exposure and bladder cancer among TT homozygotes in the same SNP. Bladder cancer cases were also 60% less likely to be homozygotes for the A allele in rs1476413 in MTHFR compared to controls (OR = 0.40; 95% CI = 0.18-0.88).

**Conclusions:**

Variation in As3MT and MTHFR is associated with bladder cancer among those exposed to relatively low concentrations of inorganic arsenic. Further investigation is warranted to confirm these findings.

## Background

Cancer of the urinary bladder (or bladder) is the seventh most common malignancy worldwide among men and the 17^th^ among women with approximately 386,000 new cases diagnosed each year [1,2]. In the United States (U.S.), bladder cancer is the fourth most common cancer diagnosed among men and the eleventh among women with a projected 73,510 new cases to be diagnosed in 2012 [3]. A vast majority of bladder tumors arise in the urothelium and the most common histology is transitional cell carcinoma (TCC).

Ingestion of inorganic arsenic primarily from groundwater supplies has been linked to a number of adverse health conditions including both benign and malignant skin lesions, peripheral vascular disease and cancers of the kidney, lung and bladder [4,5]. The data to support these relationships were first gathered from investigations in areas of chronic and endemic arseniasis, however, in the past decade there has been an increasing amount of attention devoted to study of the health consequences of low to moderate concentrations of arsenic consumed from contaminated household drinking water (<100 μg/L). While studies in Argentina, Finland and the U.S. indicate an increased risk of bladder cancer among smokers exposed to higher levels of arsenic, they do not report any increase in risk overall associated with concentrations in this range [6-9]. In a recent report, our findings from a population-based case–control study conducted in Southeastern Michigan are in agreement with other U.S. studies indicating no significant association overall between arsenic exposure from drinking water and bladder cancer risk [10]. However the limitations inherent in the design of these studies, particularly the difficulty in estimating arsenic exposure over an extended period of time to account for the long-induction latency period for cancer, introduces the possibility of exposure misclassification which may explain the null findings. Results have not been uniformly negative, as studies in Northeastern Taiwan, Chile and others in Argentina have reported increases in bladder cancer risk and mortality associated with arsenic concentrations as low as 10-50 μg/L depending upon the length of exposure [11-15].

Crucial to any investigation of an environmental exposure such as arsenic and potential health risks is the consideration of interindividual variability in its metabolism and clearance. The primary pathway for the metabolism of inorganic arsenic is methylation to monomethylated (monomethylarsonic acid [MMA^V+^, monomethylarsonous acid [MMA^III+^) and eventually dimethylated (dimethylarsinic acid [DMA^V+^, dimethylarsinous acid [DMA^III+^) forms [16]. In humans, the process is incomplete and all forms are excreted in urine in varying proportions, with arsenite and monomethylated forms considered the most bioactive and linked to enhanced risk of arsenic-induced health hazards including bladder cancer [17-20]. The typical urine profile in populations exposed to inorganic arsenic contains 10-30% inorganic arsenic, 10-20% MMA and 60-80% DMA [16]. The interindividual variation in the relative proportion of inorganic arsenic and its methylated metabolites excreted in urine has been attributed to differences in gender, age, pregnancy, smoking behavior and diet [21]. Moreover, genetics have long been suspected to play an important role in arsenic metabolism [21,22]. Polymorphisms in a number of genes have been associated with variation in methylation capacity with Glutathione S-Transferase Omega-1 (GSTO-1), Methylene-tetrahydrofolate Reductase (MTHFR) and Arsenic (+3) Methyltransferase (As3MT) as three of the more widely investigated genes [23-33].

Therefore, we set out to examine the association between specific polymorphisms in each of these genes with bladder cancer in a pilot case–control study. This study is one of the first to investigate the interaction between genes involved in arsenic metabolism and lifetime arsenic exposure on risk of bladder cancer conducted in a residentially stable population in Southeast Michigan.

## Methods

A population-based case–control study was conducted in eleven counties in Southeastern Michigan based on evidence of moderately elevated (<100 μg/L) arsenic concentrations in groundwater supplies. It has been estimated that approximately 8% of the total population in the counties from which our participants were selected are exposed to arsenic concentrations which exceed the Maximum Contaminant Level (MCL) of 10 μg/L set by the U.S. Environmental Protection Agency [34]. A detailed summary of study methods has been previously reported [10]. Eligible participants were residents of Genesee, Huron, Ingham, Jackson, Lapeer, Livingston, Oakland, Sanilac, Shiawasee, Tuscola and Washtenaw counties for at least 5 years prior to diagnosis (bladder cancer cases) and interview (controls). Bladder cancer cases diagnosed between January 1^st^, 2000 and December 31^st^, 2004 were identified from the Michigan Cancer Surveillance Program, the state’s cancer registry, within the Division of Vital Records, Michigan Department of Community Health. Cases were aged 21 to 80 years at date of diagnosis, histopathologically confirmed, with no history of invasive cancer prior to bladder cancer diagnosis. Of the 1,634 potentially eligible cases, 25% (n = 411) completed all phases of the initial study protocol. The most common reasons for non-participation were 1.) refusing permission to registry for initial release of contact information (41%); and 2.) death prior to registry contact (22%). Disease-free controls were identified through random-digit dialing and frequency matched to cases by age (± 5 years), gender and race. Among the 2,132 eligible controls identified, 27% (n = 566) completed all phases of the initial protocol.

All participants completed a computer-assisted telephone interview conducted by trained interviewers from the Michigan Public Health Institute (MPHI). The interview collected information on demographics, water and other fluid consumption both at home and away from home, a dietary questionnaire, smoking, medical and occupational history. Patterns of fluid consumption over the participant’s adult lifetime were reported, including a detailed characterization of any major changes in fluid consumption. After the initial interview, research team members scheduled a visit to the home of each participant to collect complete residential and occupational histories. Participants were asked to provide information on duration of residence/employment, drinking water source at each location, change in water source and a street address (or nearest cross streets). Each residence in the study area was geocoded and assigned a specific coordinate in ArcGIS (v.9.0; ESRI, Redlands, CA) with no geocoding of residences outside the study area. During the in-home interview, household water and biospecimens were also collected.

A genetic component to the larger case–control study was initiated in 2005, at the approximate midpoint in subject recruitment. The protocol was amended to request that participants provide a saliva specimen during their scheduled in-home visit. A total of 219 cases and 273 controls, representing ~98% of subjects recruited during this time, submitted saliva samples for genetic studies. Genomic DNA was isolated from these specimens using the Puregene DNA Purification buccal cell kit (Gentra Systems, Minneapolis, MN). All protocols were reviewed and approved by the University of Michigan Health Sciences Institutional Review Board and all participants provided written informed consent.

### Measure of lifetime arsenic exposure

Our approach for estimating average lifetime arsenic exposure and analysis of household water samples has been previously described [10,35]. Briefly, an estimation of arsenic concentrations from past residences on public well water supplies relied on data that were extracted from the Michigan state arsenic database and via the contact of specific municipality within the study area where the participant reported a prior residency. Public surface water supplies from past residences were assigned the average measured level of surface water in the study population (0.30 μg/L). For past private wells inside the study area, a geostatistical model was developed for predicting arsenic concentrations [36]. For public supplies or private wells outside of the study area, arsenic concentrations were estimated using city averages from the U.S. Geological Survey (USGS) database [37]. Estimates of average lifetime residential exposure to arsenic were constructed independent of case–control status, combining the measured concentrations from household water samples collected from the subject’s current residence and data gathered from past residences with the assumption that arsenic concentrations were relatively stable within a particular well. This assumption is supported by our own internal investigation [38] and in the literature [39]. The measure was then weighted by duration of residence with an average (in μg/L) calculated across the adult lifetime [10]. The household water samples were analyzed for arsenic concentration using an inductively coupled plasma mass spectrometry unit (ICP-MS, Model 7500c, Agilent Technologies, Santa Clara, CA) at the University of Michigan.

### SNP selection and genotyping methods

A total of twelve single nucleotide polymorphisms (SNPs) in GSTO-1 (rs4925, rs2282326), As3MT (rs1046778, rs11191438, rs11191439, rs3740400, rs7085104) and MTHFR (rs1801131, rs1801133, rs6541003, rs9651118, rs4846048, rs1476413) were genotyped using Taqman SNP genotyping assays (Applied Biosystems, Foster City, CA). These SNPs were selected based upon previous citation/functional relevance in the literature, **or** a minor allele frequency of ≥ 10% among CEPH (Centre d’Etude du Polymorphisme Humain) Utah European ancestry samples (CEU) in HapMap and an ability to tag major haplotype blocks within each gene in the same samples [40]. We used the ABI PRISM 7900HT Sequence Detection System and the SDS software (Applied Biosystems, version 2.1) to distinguish SNP alleles. PCR primers were purchased from Invitrogen Life Technologies (Carlsbad, California) and are available upon request. Each PCR reaction contained 25μL total volume (2.5μL of 10X PCR Buffer (Invitrogen Life Technologies, Inc.), 0.5 μL 50 mM MgCl_2_, 0.5μL 10 mM dNTPs, 2.5 μL each of the two PCR primers at 5 μM concentration, 2 μL of template DNA at 20 ng/μL, 0.125 μL of PlatinumTaq Polymerase (Invitrogen), and 14.375 μL of H_2_0. PCR products were cleaned using MinElute PCR purification kit (Germantown, Massachusetts) and sequenced using an ABI prism 3100 Genetic Analyzer using Big Dye Terminator v1.1 chemistries (Applied Biosystems, Foster City, California). The average genotyping call rate was 97.5%, with call rates ≥ 94.6% for each SNP. Undetermined samples from an assay were sequenced if DNA was available, resulting in a final genotyping call rate of 99.5%, with call rates ≥ 98.8% for each SNP. A random subset of samples (2%) were also duplicated and verified by direct sequencing without discrepancy.

### Statistical methods

Statistical analyses were performed using Statistical Analysis Software (SAS Institute Inc. v. 9.1, Cary, N.C.). All tests were evaluated using a two-sided hypothesis test with statistical significance interpreted as having a p-value less than or equal to 0.05. Differences in age, gender, race, smoking history, average arsenic exposure and the distribution of genotypes for each SNP between bladder cancer cases and controls were tested using either the Wilcoxon rank-sum test or a simple chi-square test. For each SNP, we calculated the minor allele frequency among controls (Table 1).

**Table 1:** Single nucleotide polymorphisms in Glutathione S-Transferase Omega, Arsenic Methyltransferase and 5,10 Methylene-tetrahydrofolate Reductase

**SNP‡**	**Gene**	**Major/Minor Allele†**	MAF (%)*
rs4925	GSTO-1	*C/A (Ala140Asp)*	32.5
rs2282326	GSTO-1	*A/C*	38.9
rs1046778	As3MT	*T/C*	29.9
rs3740400	As3MT	*A/C*	35.8
rs11191439	As3MT	*T/C (Met287Thr)*	9.2
rs11191438	As3MT	*G/C*	39.6
rs7085104	As3MT	*A/G*	33.9
rs1801131	MTHFR	*A/C (Glu429Ala)*	33.0
rs1801133	MTHFR	*C/T (Ala222Val)*	33.5
rs6541003	MTHFR	*A/G*	42.7
rs9651118	MTHFR	T/C	19.1
rs4846048	MTHFR	*A/G*	30.8
rs1476413	MTHFR	*G/A*	29.4

* Minor Allele Frequency (%) among controls.

† Non-synonymous SNPs denoted with amino acid substitution.

‡ SNP = single nucleotide polymorphism.

The observed genotype distribution for each SNP among controls was tested for consistency with HWE expectations using Pearson’s chi-square test. Linkage disequilibrium (LD) between selected SNPs within each gene was evaluated using the PLINK software package (http://pngu.mgh.harvard.edu/purcell/plink)[41] Unconditional logistic regression was used to determine whether genotypes within each SNP were associated with bladder cancer under the log-additive, dominant and recessive models. A post-hoc test comparing homozygotes for the minor allele and heterozygotes each to homozygotes for the major allele (the referent) was also performed. Multivariable models simultaneously adjusted for the effect of each SNP, age (continuous), and smoking history (ever/never). Because adjustment for race and gender did not alter the odds ratios, they were not included in the final models. The joint effect of each SNP and arsenic concentration was evaluated by including an interaction term in the regression models which combined genotype (dominant model) and average arsenic concentration measured continuously. Additional analyses stratified by genotype were performed, calculating odds ratios for bladder cancer associated with arsenic measured both continuously and according to quartiles in the distribution of exposure among controls. The p-values presented in the tables correspond to the β coefficient for the interaction term. Potential haplotypes for As3MT were constructed from genotype data and differences in haplotype frequency (excluding rare haplotypes [<2%]) between cases and controls were evaluated using the Haplo.stat software package http://mayoresearch.mayo.edu/mayo/research/schaid_lab/software.cfm. The same package was used to estimate the association between haplotype (under a log-additive model) and bladder cancer, adjusting for age and smoking history [42].

## Results

Relevant characteristics of the 219 bladder cancer cases and 273 controls participating in the pilot investigation are summarized in Table 2. By design, cases and controls were similar with respect to age, race and gender. Approximately 75% of participants were men, 95% were white and the mean age at diagnosis (among cases) and at interview (among controls) was 66 years (standard deviation ± 9.5 years). Bladder cancer cases were more likely to report that they had either formerly smoked or were current smokers at the time of diagnosis compared to controls at time of interview (78% versus 53%; p < 0.0001). Furthermore, the estimated mean number of pack-years among smokers was higher among cases than controls. As has been reported in the larger case–control study, there was no significant difference between genotyped cases and controls in lifetime average exposure to arsenic (p = 0.45) with an estimated median concentration of 1.36 μg/L (interquartile range = 3.33 μg/L) in the study population.

**Table 2:** Characteristics of Study Participants

**Characteristic**	**Bladder Cancer Cases n = 219**	**Controls n = 273**	p-value*
Age (in years)†	66.5 (+/− 9.4)	66.7 (+/− 10.0)	0.85
Gender			
Males	169 (77.2%)	195 (71.4%)	
Females	50 (23.8%)	78 (28.6%)	0.15
Race			
White	206 (94.1%)	260 (95.1%)	
Black	5 (2.3%)	2 (0.8%)	
Other	8 (3.7%)	11 (4.0%)	0.35
Smoking			
Ever smoked	172 (78.5%)	146 (53.5%)	
Never smoked	47 (21.5%)	127 (46.5%)	<0.0001*
Pack years smoked †∞	33.2 (+/− 22.7)	24.4 (+/− 19.8)	0.003*
Arsenic concentration in water (TWA) in μg/L**			
	1.31 (3.37)	1.44 (3.27)	0.45

* Derived from either Chi-square or Wilcoxon-Rank Sum test.

TWA = Time-Weighted Average.

† Mean (± standard deviation).

∞ Among smokers.

** Median (interquartile range).

The genotype distribution observed for each of the tested SNPs among controls was consistent with HWE. Table 3 summarizes the findings of our post-hoc testing of associations between genotype for each SNP and bladder cancer risk adjusted for age and smoking history, however this adjustment produced little difference from the crude estimates of risk. We did not observe any significant associations between SNPs in GSTO-1, As3MT and MTHFR and bladder cancer risk overall with one exception. Bladder cancer cases were 60% less likely to be homozygotes for the A allele in rs1476413 in MTHFR compared to controls (OR = 0.40; 95% CI = 0.18-0.88).

**Table 3:** Odds of bladder cancer by genotype in genes of interest (Adjusted Odds Ratios and 95% Confidence Intervals)

**SNP**	**Genotype**	**Cases N (%)**	**Controls N (%)**	**Age and smoking-adjusted OR (95% CI)**
**GSTO-1**				
rs4925	C/C	97 (44.7)	123 (46.1)	1.00
	C/A	98 (45.2)	113 (42.3)	1.14 (0.77 – 1.68)
	A/A	17 (7.8)	30 (11.2)	0.82 (0.42 – 1.61)
	C/C			1.00
	C/A & A/A			1.07 (0.74 – 1.56)
rs2282326	A/A	84 (39.1)	104 (38.5)	1.00
	A/C	97 (44.3)	122 (45.2)	1.03 (0.68 – 1.55)
	C/C	34 (15.8)	44 (16.3)	1.07 (0.61 – 1.86)
	A/A			1.00
	A/C & C/C			1.04 (0.71 – 1.52)
**As3MT**				
rs1046778	T/T	112 (52.1)	134 (49.3)	1.00
	T/C	80 (37.2)	113 (41.5)	0.83 (0.56 – 1.24)
	C/C	23 (11.4)	25 (9.2)	1.00 (0.53 – 1.91)
	T/T			1.00
	T/C & C/C			0.87 (0.60 – 1.26)
rs3740400	A/A	80 (37.0)	113 (41.7)	1.00
	A/C	118 (54.6)	122 (45.0)	1.34 (0.90 – 2.00)
	C/C	18 (8.3)	36 (13.3)	0.71 (0.37 – 1.36)
	A/A			1.00
	A/C & C/C			1.20 (0.82 – 1.75)
rs11191439	T/T	172 (80.0)	222 (82.2)	1.00
	T/C	39 (18.1)	46 (17.0)	1.12 (0.69 – 1.82)
	C/C	4 (1.9)	2 (0.7)	2.75 (0.47 – 16.77)
	T/T			
	T/C & C/C			1.18 (0.74 – 1.90)
rs11191438	G/G	71 (32.7)	103 (37.9)	1.00
	G/C	113 (52.1)	122 (44.9)	1.26 (0.84 – 1.90)
	C/C	33 (15.2)	47 (17.3)	1.00 (0.57 – 1.74)
	G/G			1.00
	G/C & C/C			1.19 (0.81 – 1.76)
rs7085104	A/A	86 (39.5)	119 (43.9)	1.00
	A/G	112 (51.4)	120 (44.3)	1.28 (0.87 – 1.90)
	G/G	20 (9.2)	32 (11.8)	0.88 (0.46 – 1.68)
	A/A			
	A/G & G/G			1.20 (0.82 – 1.74)
**MTHFR**				
rs1801131	A/A	95 (43.6)	127 (46.7)	1.00
	C/A	109 (50.0)	111 (40.8)	1.39 (0.94 – 2.05)
	C/C	14 (6.4)	34 (12.5)	0.55 (0.28 – 1.11)
	A/A			1.00
	C/A & C/C			1.19 (0.82 – 1.72)
rs1801133	C/C	92 (42.0)	121 (44.3)	1.00
	C/T	106 (48.4)	121 (44.3)	1.18 (0.80 – 1.75)
	T/T	21 (9.6)	31 (11.4)	0.86 (0.45 – 1.62)
	C/C			1.00
	C/T & T/T			1.11 (0.77 – 1.62)
rs6541003	A/A	69 (31.7)	89 (32.8)	1.00
	A/G	125 (57.3)	131 (48.3)	1.36 (0.91 – 2.10)
	G/G	24 (11.0)	51 (18.8)	0.65 (0.36 – 1.18)
	A/A			1.00
	A/G & G/G			1.17 (0.79 – 1.75)
rs9651118	T/T	138 (63.0)	180 (65.9)	1.00
	C/T	68 (31.1)	83 (30.4)	1.03 (0.69 – 1.54)
	C/C	13 (5.9)	10 (3.7)	1.54 (0.63 – 3.71)
	T/T			1.00
	C/T & C/C			1.08 (0.74 – 1.59)
rs4846048	A/A	102 (47.0)	131 (49.2)	1.00
	A/G	100 (46.1)	111 (40.8)	1.25 (0.85 – 1.85)
	G/G	15 (6.9)	30 (11.0)	0.66 (0.33 – 1.32)
	A/A			
	A/G & G/G			1.12 (0.78 – 1.63)
rs1476413	G/G	120 (55.1)	139 (51.3)	1.00
	G/A	88 (40.4)	105 (38.8)	1.01 (0.69– 1.49)
	A/A	10 (4.6)	27 (10.0)	0.40 (0.18 – 0.88)
	G/G			1.00
	G/A & A/A			0.88 (0.61 – 1.27)

‡ Models simultaneously adjusted for SNP, age (continuous), smoking history (ever/never).

We then stratified the study population according to genotype, examining the differences in the distribution between time-weighted average (TWA) of arsenic exposure (divided into quartiles and measured continuously) between cases and controls (Tables 4 and 5). There was evidence for interaction between several of the SNPs in As3MT and arsenic exposure. Among persons with one or more copies of the C allele in rs11191439 (Met287Thr), higher average arsenic exposure was associated with an increase in the odds of bladder cancer (OR = 1.17; 95% CI = 1.04-1.32 per 1 μg/L increase). However, no association was observed between average arsenic exposure and bladder cancer among individuals who were TT homozygotes for the same SNP. This same polymorphism was also associated with an increase in the odds of bladder cancer among all study subjects, however the estimate was not statistically significant (OR = 2.75; 95%CI = 0.47-16.77 among CC homozygotes). Interactions with SNPs rs3740400, rs11191438 and rs7085104 and arsenic exposure were also observed (Table 4). These SNPs were in strong LD with one another (r^2^ values ranging from 0.83-0.95) ( Additional file 1: Table S1). And while there was nominal evidence for interaction with the rs4925 SNP in GSTO-1 and arsenic exposure, we not find evidence for interaction with selected SNPs in MTHFR and arsenic (Table 5). Haplotype-based association analyses for the As3MT gene were consistent with the single SNP results (Table 6). No evidence for an association between haplotypes in the As3MT gene and bladder cancer was detected when using the entire sample of participants. There was, however, an association between the As3MT haplotype which included the Met287Thr polymorphism (compared to the most common or referent haplotype) and bladder cancer for participants exposed to arsenic concentrations higher than 3.72 μg/L (the uppermost quartile) (OR = 3.90; 95% CI = 1.39-10.9), but not among those exposed to lower concentrations.

**Table 4:** Odds of bladder cancer associated with As3MT SNPs and arsenic from drinking water (in quartiles†)

**SNP**	**Major allele homozygote**			**Minor allele carrier**			
	Cases n (%)	Controls n (%)	Age and smoking adjusted OR (95% CI)	Cases n (%)	Controls n (%)	Age and smoking adjusted OR (95% CI)	p-interaction
**As3MT**							
**rs1046778**							
Q1	30 (26.8)	33 (24.6)	1.00	30 (29.1)	35 (25.4)	1.00	
Q2	29 (25.9)	30 (22.4)	0.93 (0.44 - 1.97)	23 (22.3)	39 (28.3)	0.71 (0.34 – 1.46)	
Q3	25 (22.3)	34 (25.4)	0.81 (0.38 - 1.71)	23 (22.3)	34 (24.6)	0.77 (0.37 - 1.61)	
Q4	28 (25.0)	37 (27.6)	0.74 (0.36 - 1.56)	27 (26.2)	30 (21.7)	1.14 (0.55 - 2.36)	
Continuous‡	112 (45.5)	134 (54.5)	1.00 (0.95 – 1.05)	103 (42.7)	138 (57.3)	1.03 (0.99 – 1.08)	0.33
**rs3740400**							
Q1	23 (28.8)	19 (16.8)	1.00	38 (27.9)	49 (31.0)	1.00	
Q2	24 (30.0)	28 (24.8)	0.60 (0.25 - 1.43)	28 (20.6)	41 (25.9)	0.87 (0.45 - 1.69)	
Q3	18 (22.5)	27 (23.9)	0.55 (0.22 - 1.36)	30 (22.1)	40 (25.3)	0.94 (0.49 - 1.82)	
Q4	15 (18.8)	39 (34.5)	0.24 (0.10 - 0.60)	40 (29.4)	28 (17.7)	2.08 (1.06 – 4.07)	
Continuous	80 (41.5)	113 (58.6)	0.96 (0.91 – 1.02)	136 (46.3)	158 (53.7)	1.08 (1.02 – 1.14)	0.006
**rs11191439**							
Q1	48 (27.9)	46 (20.7)	1.00	13 (30.2)	21 (43.8)	1.00	
Q2	44 (25.6)	57 (25.7)	0.71 (0.40 - 1.28)	7 (16.3)	11 (22.9)	0.96 (0.27 – 3.37)	
Q3	39 (22.7)	58 (26.1)	0.62 (0.34 - 1.12)	9 (20.9)	10 (20.8)	1.68 (0.49 – 5.75)	
Q4	41 (23.8)	61 (27.5)	0.61 (0.34 - 1.09)	14 (32.6)	6 (12.5)	6.88 (1.72 – 27.6)	
Continuous	172 (43.7)	222 (56.4)	1.00 (0.97 - 1.04)	43 (47.3)	48 (52.8)	1.17 (1.04 – 1.32)	0.02
**rs11191438**							
Q1	19 (26.8)	19 (18.4)	1.00	42 (28.8)	49 (29.0)	1.00	
Q2	22 (31.0)	23 (22.3)	0.72 (0.28 - 1.84)	30 (20.5)	46 (27.2)	0.79 (0.42 - 1.49)	
Q3	16 (22.5)	29 (28.2)	0.49 (0.19 - 1.26)	33 (22.6)	39 (23.1)	0.98 (0.52 - 1.86)	
Q4	14 (19.7)	32 (31.1)	0.28 (0.10 - 0.74)	41 (28.1)	35 (20.7)	1.60 (0.85 – 3.03)	
Continuous	71 (40.8)	103 (59.2)	0.96 (0.90 – 1.03)	146 (46.4)	169 (53.7)	1.04 (1.00 – 1.09)	0.04
**rs7085104**							
Q1	25 (29.1)	20 (16.8)	1.00	35 (26.5)	48 (31.6)	1.00	
Q2	26 (30.2)	30 (25.2)	0.59 (0.26 - 1.36)	28 (21.2)	39 (25.7)	0.99 (0.50 - 1.95)	
Q3	20 (23.3)	28 (23.5)	0.57 (0.24 - 1.37)	29 (22.0)	39 (25.7)	0.99 (0.50 - 1.93)	
Q4	15 (17.4)	41 (34.5)	0.23 (0.09 - 0.55)	40 (30.3)	26 (17.1)	2.46 (1.23 – 4.92)	
Continuous	86 (42.0)	119 (58.1)	0.96 (0.91-1.01)	132 (46.5)	152 (53.5)	1.09 (1.03 – 1.16)	0.002

† Quartile distribution of average arsenic exposure for among all genotyped participants.

Q1 = 1^st^ quartile (< 0.55 μg/L).

Q2 = 2^nd^ quartile (0.55-1.45 μg/L).

Q3 = 3^rd^ quartile (1.45-3.72 μg/L).

Q4 = 4^th^ quartile (>3.72 μg/L).

‡ Odds Ratio per one unit increase in the continuous measure of lifetime arsenic exposure by genotype adjusted for age and smoking history (ever/never).

**Table 5:** Odds of bladder cancer associated with GSTO-1 and MTHFR SNPs and arsenic from drinking water†

**SNP**	**Major allele homozygote**			**Minor allele carrier**			
	Cases n (%)	Controls n (%)	Age and smoking adjusted OR (95% CI)	Cases n (%)	Controls n (%)	Age and smoking adjusted OR (95% CI)	p-interaction
**GSTO-1**							
**rs4925**							
Q1	29 (29.9)	31 (25.2)	1.00	29 (25.2)	37 (25.9)	1.00	
Q2	22 (22.7)	34 (27.6)	0.71 (0.33 – 1.54)	28 (24.3)	34 (23.8)	1.00 (0.49 – 2.05)	
Q3	23 (23.7)	34 (27.6)	0.71 (0.33 – 1.52)	26 (22.6)	30 (21.0)	1.12 (0.53 – 2.34)	
Q4	23 (23.7)	24 (19.5)	1.23 (0.55 – 2.76)	32 (27.8)	42 (29.4)	0.90 (0.45 – 1.80)	
Continuous	97 (44.1)	123 (55.9)	1.10 (1.02 – 1.18)	115 (44.6)	143 (55.4)	1.00 (0.96 – 1.03)	0.03
**rs2282326**							
Q1	27 (32.1)	22 (21.2)	1.00	32 (24.4)	46 (27.7)	1.00	
Q2	21 (25.0)	30 (28.8)	0.63 (0.28 – 1.44)	32 (24.4)	39 (23.5)	1.08 (0.55 – 2.11)	
Q3	18 (21.4)	29 (27.9)	0.49 (0.21 – 1.13)	30 (22.9)	38 (22.9)	1.17 (0.59 – 2.32)	
Q4	18 (21.4)	23 (22.1)	0.76 (0.32 – 1.84)	37 (28.2)	43 (25.9)	1.13 (0.59 – 2.17)	
Continuous	84 (44.7)	104 (55.3)	1.06 (0.99 – 1.14)	131 (44.1)	166 (55.9)	1.01 (0.97 – 1.04)	0.21
**MTHFR**							
**rs1801131**							
Q1	27 (28.4)	34 (26.8)	1.00	34 (27.6)	34 (23.4)	1.00	
Q2	23 (24.2)	42 (33.1)	0.68 (0.32 – 1.42)	30 (24.4)	27 (18.6)	1.07 (0.51 – 2.24)	
Q3	23 (24.2)	25 (19.7)	1.04 (0.48 – 2.27)	26 (21.1)	43 (29.7)	0.64 (0.32 – 1.30)	
Q4	22 (23.2)	26 (20.5)	1.00 (0.46 – 2.20)	33 (26.8)	41 (28.3)	0.86 (0.43 – 1.72)	
Continuous	95 (42.8)	127 (57.2)	1.01 (0.96 – 1.05)	123 (45.9)	145 (54.1)	1.03 (0.99-1.08)	0.43
**rs1801133**							
Q1	26 (28.3)	29 (24.0)	1.00	35 (27.6)	39 (25.7)	1.00	
Q2	25 (27.2)	28 (23.1)	1.03 (0.47 – 2.22)	29 (22.8)	41 (27.0)	0.70 (0.35 – 1.41)	
Q3	19 (20.7)	36 (29.8)	0.65 (0.30 – 1.42)	30 (23.6)	32 (21.1)	0.89 (0.44 – 1.81)	
Q4	22 (23.9)	28 (23.1)	0.92 (0.42 – 2.02)	33 (26.0)	40 (26.3)	0.85 (0.43 – 1.69)	
Continuous	92 (43.2)	121 (56.8)	1.07 (1.00 – 1.14)	127 (45.5)	152 (54.5)	0.99 (0.96 – 1.03)	0.05
**rs6541003**							
Q1	17 (24.6)	22 (24.7)	1.00	44 (29.5)	45 (24.7)	1.00	
Q2	16 (23.2)	29 (32.6)	0.72 (0.29 – 1.79)	37 (24.8)	40 (22.0)	0.89 (0.47 – 1.67)	
Q3	18 (26.1)	19 (21.3)	1.06 (0.42 – 2.69)	31 (20.8)	49 (26.9)	0.68 (0.36 – 1.28)	
Q4	18 (26.1)	19 (21.3)	1.20 (0.47 – 3.04)	37 (24.8)	48 (26.4)	0.80 (0.43 – 1.49)	
Continuous	69 (43.7)	89 (56.3)	1.01 (0.96 – 1.05)	149 (45.0)	182 (55.0)	1.03 (0.98 – 1.08)	0.52
**rs9651118**							
Q1	37 (26.8)	45 (25.0)	1.00	24 (29.6)	23 (24.7)	1.00	
Q2	37 (26.8)	46 (25.6)	0.90 (0.48 – 1.70)	17 (21.0)	23 (24.7)	0.73 (0.30 – 1.77)	
Q3	32 (23.2)	42 (23.3)	0.88 (0.46 – 1.69)	17 (21.0)	26 (28.0)	0.66 (0.28 – 1.57)	
Q4	32 (23.2)	47 (26.1)	0.84 (0.44 – 1.60)	23 (28.4)	21 (22.6)	1.01 (0.43 – 2.38)	
Continuous	138 (43.4)	180 (56.6)	1.01 (0.97 – 1.06)	81 (46.6)	93 (53.5)	1.02 (0.97 – 1.08)	0.73
**rs4846048**							
Q1	25 (24.5)	31 (23.7)	1.00	36 (31.3)	37 (26.2)	1.00	
Q2	23 (22.5)	37 (28.2)	0.74 (0.34 – 1.59)	29 (25.2)	32 (22.7)	0.91 (0.45 – 1.84)	
Q3	25 (24.5)	29 (22.1)	0.88 (0.41 – 1.93)	24 (20.9)	39 (27.7)	0.73 (0.36 – 1.49)	
Q4	29 (28.4)	34 (26.0)	0.97 (0.46 – 2.05)	26 (22.6)	33 (23.4)	0.89 (0.44 – 1.83)	
Continuous	102 (43.8)	131 (56.2)	1.01 (0.97 – 1.06)	115 (44.9)	141 (55.1)	1.03 (0.97 – 1.09)	0.65
**rs1476413**							
Q1	30 (25.0)	32 (23.0)	1.00	31 (31.6)	35 (26.5)	1.00	
Q2	29 (24.2)	46 (33.1)	0.66 (0.33 – 1.34)	24 (24.5)	23 (17.4)	1.14 (0.52 – 2.52)	
Q3	32 (26.7)	27 (19.4)	1.20 (0.58 – 2.51)	17 (17.3)	41 (31.1)	0.48 (0.22 – 1.04)	
Q4	29 (24.2)	34 (24.5)	0.87 (0.42 – 1.80)	26 (26.5)	33 (25.0)	0.96 (0.46 – 2.01)	
Continuous	120 (46.3)	139 (53.7)	1.02 (0.98 – 1.06)	98 (42.6)	132 (57.4)	1.02 (0.96 – 1.09)	0.93

† Quartile distribution of average arsenic exposure for among all genotyped participants.

Q1 = 1^st^ quartile (< 0.55 μg/L).

Q2 = 2^nd^ quartile (0.55-1.45 μg/L).

Q3 = 3^rd^ quartile (1.45-3.72 μg/L).

Q4 = 4^th^ quartile (>3.72 μg/L).

‡ Odds Ratio per one unit increase in the continuous measure of lifetime arsenic exposure by genotype adjusted for age and smoking history (ever/never).

**Table 6:** Common haplotypes in the As3MT gene and bladder cancer by average level of arsenic from drinking water

	**All Subjects**			*As ≤3.72 μg/L			**As >3.72 μg/L**		
Haplotype^**^	Cases (%)	Controls (%)	OR‡† (95% CI)	Cases (%)	Controls (%)	OR (95% CI)	Cases (%)	Controls (%)	OR (95% CI
T-T-G-T-A	57.5	58.3	1.00	58.1	55.2	1.00	55.4	67.5	1.00
C-G-C-T-G	22.0	22.7	0.97 (0.71 – 1.34)	20.8	24.3	0.78 (0.53 – 1.13)	25.4	17.9	1.92 (0.98 – 3.76)
T-G-C-C-G	11.1	9.3	1.20 (0.78 – 1.84)	9.9	11.0	0.86 (0.52 – 1.41)	14.5	4.4	3.90 (1.39 – 10.9)
C-T-C-T-A	6.0	5.5	1.14 (0.64 – 2.01)	7.1	5.3	1.42 (0.75 – 2.69)	2.8	6.4	0.54 (0.13 – 2.22)

* Time-weighted average arsenic (As) concentration across lifecourse; dichotomized at the approximate 75% percentile in.

distribution among controls.

† OR = odds ratio CI==confidence interval.

‡ Adjusted for age and smoking history.

** Order of 5 As3MT SNPs in haplotype models (rs1046778, rs3740400, rs11191438, rs11191439, rs7085104).

## Discussion

In 2006, the U.S. Environmental Protection Agency lowered the MCL for arsenic concentrations in drinking water provided by public water supplies in the U.S. from 50 μg/L to 10 μg/L, in response to growing concern and mounting evidence that inorganic arsenic was hazardous to human health at relatively low levels [4,43]. Results from studies conducted across several regions in the U.S. generally do not indicate these levels elevate risk of bladder cancer, however some studies report elevated risks among certain subgroups in the population (i.e. smokers) in conjunction with low-level arsenic exposure[6,8-10]. Findings from the current investigation suggest that possessing one or more copies of the Met287Thr polymorphism in the As3MT gene (rs11191439), coupled with higher average arsenic exposure is associated with an increase in bladder cancer risk. Our study population had levels of arsenic primarily below the current MCL, therefore the findings pertain to potential genetic susceptibility to effects of arsenic at relatively low-levels.

While our study is the first to report an interaction between As3MT (10q24.32) and arsenic exposure on bladder cancer risk, these findings are consistent with evidence gleaned from several other studies demonstrating that polymorphisms in As3MT are involved in the methylation of inorganic arsenic [24-29,33,44]. A study of urinary arsenic species in a Mexican population exposed to arsenic at concentrations averaging 5 μg/L to 43 μg/L was the first to report that three polymorphic sites in As3MT were associated with a higher ratio of DMA^V^ to MMA^V^, with a single polymorphism (rs11191453) also associated with a higher ratio of arsenite to MMA^V^ in children, but not adults [33]. Subsequent studies have pointed to the Met287Thr polymorphism as being associated with enhanced As3MT enzymatic activity [45] and a higher percentage of MMA to DMA in urine samples among individuals carrying at least one copy of the variant (C) allele [24,29,44,46]. Furthermore, the findings of Lindberg et al., suggest that the influence of the Met387Thr polymorphism is greater among men than women explaining approximately 20% of the variation in methylation ability in men, but just 4% in women [47]. In a recent report, no association was observed between any of eight tested SNPs in As3MT and bladder cancer overall and no clear evidence of effect modification by A3sMT genotype. However, despite the study’s limited statistical power to address interaction between arsenic exposure and genotype, a number of the odds ratios reported among participants exposed to higher arsenic concentrations were elevated, but with wide confidence intervals [48].

Gomez-Rubio and colleagues caution against attributing enhanced arsenic methylation to any single SNP in As3MT, or for that matter any one gene, as they identified a large (347 kb) region in strong LD including four genes in addition to As3MT [49]. We observed strong pairwise LD between three of four As3MT polymorphisms interacting with arsenic exposure ( Additional file 1: Table S1). However, the Met287Thr polymorphism did not strongly co-segregate with the other tested SNPs. Our results suggest that homozygotes for the more common allele in each of these SNPs (rs3740400, rs11191438 and rs7085104) had a lower risk of bladder cancer in conjunction with arsenic exposure, however minor allele carriers were at an increased risk. Prior investigations of rs3740400, or those observed in LD with this particular SNP, suggest an altered DMA to MMA ratio associated with the minor allele [28,29,50]. However, the observed racial and ethnic variation in allele frequency and patterns of LD, coupled with the worldwide geographic variability in not only exposure to arsenic but other risk factors, make the comparison between previous findings and our own difficult and an area deserving further investigation.

There was some evidence, albeit weak, to support an interaction between the rs4925 SNP in GSTO-1 and arsenic exposure in this investigation. Specifically, there was evidence of a positive association between arsenic exposure and bladder cancer among those homozygous wildtype for the GSTO-1 polymorphism that was not present for those with at least one variant allele. GSTO-1, also on chromosome 10 (10q25.1), has demonstrated the ability to catalyze the reduction of MMA^V^ to MMA^III^, considered the rate-limiting step in arsenic biotransformation [51]. A strong pattern of LD has been demonstrated in European Americans among the more common (MAF > 10%) polymorphisms identified [32]. The Ala140Asp (rs4925) polymorphism in GSTO-1 has been the most widely-studied, linked to risk of pre-cancerous skin lesions and cancer in arsenic-exposed populations [26,52,53], however studies have not confirmed any association between specific variants in GSTO-1 with the relative proportion of arsenic and its metabolites [47,52,54].

A greater proportion of controls compared to cases were homozygotes for a single polymorphism in MTHFR (rs1476413). The function, if any, of this particular SNP is unknown, however other polymorphisms in the MTHFR gene have been linked to a number of cancers [55]. Findings with respect to bladder cancer have been mixed [23,31,56-59]. Methylene-tetrahydrofolate reductase is an enzyme which plays a notable role in the metabolism of folate, and also considered important in arsenic methylation [60]. The 677 C → T (Ala222Val) and 1298 A → C (Glu429Ala) polymorphisms in MTHFR have been investigated relative to their influence on arsenic methylation capacity suggesting that homozygotes for the variant T allele in MTHFR 677 excrete a smaller proportion of DMA to inorganic arsenic in their urine and the opposite effect among homozygotes for the variant C allele in MTHFR 1298 compared to homozygotes for the wild-type for each SNP [23,47]. We did not find an association with either of these polymorphisms in our investigation.

Consideration of potential sources of bias is important in interpreting the findings from this investigation and a description of potential bias in the larger case–control study has been previously published [10]. The participation rate was relatively low (~25%) introducing the possibility of selection bias. To address this, we compared known characteristics of participating cases and potentially eligible, non-participating cases and found no difference between the two groups with respect to race, gender and urban versus rural residency. However, because participating cases were more likely to be diagnosed with less invasive disease and at a younger age compared to non-participating cases, our findings might not be generalizable to older patients diagnosed with advanced bladder cancer. As nearly one-quarter of potentially eligible cases died prior to registry contact, survival bias is a consideration. Deceased cases were more likely than participating cases to reside in rural areas versus urban areas at time of diagnosis (10% versus 6%). As rural areas tend to have generally higher and more variable levels of arsenic exposure, it is possible that arsenic exposure may be associated with more aggressive disease and earlier death producing a bias for the overall association between arsenic exposure and bladder cancer toward the null. However, published findings to support this notion are inconsistent [61-63]. The reasons for non-participation among eligible controls were unavailable; however an evaluation of participating controls suggested that they were geographically representative of the general population of the study area and by design similar in distribution to the cases with respect to age, gender and race. A more detailed description of this evaluation and its findings has been previously reported [10].

Lastly, because the genetic component did not begin until the midpoint in recruitment, genetic material was collected on just 492 (50.4%) of all study participants. A comparison of characteristics between participants with and without DNA found no difference between these groups by gender or smoking history (data not shown). A higher proportion of participants with DNA were white (95%) compared to those without DNA (89%) and exposed to higher arsenic concentrations (median concentration among those with DNA 1.39 μg/L compared to 1.00 μg/L among those without). However, the differences were similar between cases and controls and were a reflection of the overall composition of the study participants recruited in the latter half of the overall investigation. Nearly all (~98%) subjects recruited submitted a saliva specimen during their scheduled in-home visit.

Misclassification of lifetime arsenic exposure is a potential source of bias and may have been introduced from either inaccurate recall of residential history by participants or lack of information on groundwater arsenic concentrations for past residences on private wells. However, one of the strengths of the study was the residential stability of our population. On average, 80% of the person-years used to construct the measure of average arsenic exposure were spent in southeastern Michigan, with just 5% of reported residential histories among subjects on private wells outside of the study area and inadequate for geocoding. We do not anticipate that these sources of misclassification would differ systematically by genotype or disease status and would therefore tend to bias our results toward the null. Lastly, the study was of limited power to detect modest differences between genotype and bladder cancer risk particularly when the “at-risk” allele frequency was less common. And in the case of Met287Thr, imprecision in the estimate of risk associated with higher arsenic exposure due to the small number of exposed cases and controls. The number of participants with arsenic levels greater than the current MCL was prohibitively small to examine gene-environment interaction at arsenic concentrations greater than 10 μg/L.

## Conclusions

Results from this pilot investigation suggest that variation in As3MT and MTHFR is associated with bladder cancer among those exposed to relatively low arsenic concentrations. The Met287Thr polymorphism in As3MT, in particular, was associated with an increased risk of bladder cancer among participants exposed to higher levels of arsenic exposure. Interactions between several other SNPs in As3MT and arsenic exposure were also observed; however LD in the region makes it difficult to attribute findings to any single SNP. The underlying mechanism may also involve other genes in close proximity to As3MT. Future studies in larger populations are clearly warranted to confirm these findings as well as to broaden the exploration of genes to their potential association with arsenic in bladder cancer. If validated, these findings highlight the importance of gene-environment interaction in the evaluation of arsenic-mediated health risks in populations exposed to even low levels of arsenic.

## Abbreviations

Ala, Alanine; As3MT, Arsenic (3+) Methyltransferase; Asp, Aspartic Acid; DMA, Dimethylated arsenic; DNA, Deoxyribonucleic acid; Glu, Glutamic Acid; GSTO1, Glutathione S-Transferase Omega 1; HWE, Hardy-Weinberg Equilibrium; LD, Linkage Disequilibrium; MAF, Minor allele frequency; Met, Methionine; MgCl2, Magnesium Choride; MPHI, Michigan Public Health Institute; MTHFR, Methylene-tetrahydrofolate Reductase; MCL, Maximum Contaminant Level; MMA, Monomethylated arsenic; OR, Odds Ratio; PCR, Polymerase chain reaction; SNP, Single Nucleotide Polymorphism; TCC, Transitional Cell Carcinoma; Thr, Threonine; TWA, Time weighted average; US, United States; Val, Valine; μL, Microliters; μg/L, Micrograms per liter.

## Competing interests

The authors declare they have no competing interests.

## Authors’ contributions

JBD participated in the design and conception of the study, selection of genes, and collection of saliva specimens (with JN and JM), provided guidance during analyses and was primarily responsible for manuscript preparation. PI, SM and KA were responsible for the isolation of DNA from specimens, genotyping and sample quality control procedures. GK and EML were responsible for the conduct of the statistical analyses. AGS, JN, JM, DS, KA participated in the design and conception of the study as well as manuscript preparation and editing. KA supervised all of the genotyping efforts in her lab. All authors have read and approved of final manuscript.

## Supplementary Material

Additional file 1 **Table S1.** Pairwise linkage disequilibrium analysis of 5 selected single nucleotide polymorphisms in As3MT among participants of University of Michigan Bladder Cancer Study. Click here for file
