# Supplementary material for: Genetic variation in Glutathione S-Transferase Omega-1, Arsenic Methyltransferase and Methylene-tetrahydrofolate Reductase, arsenic exposure and bladder cancer: a case–control study
Source: Environ Health. 2012 Jun 29;11:43. doi: 10.1186/1476-069X-11-43 (PMC3465173; doi:10.1186/1476-069X-11-43)
Supplement: Additional file 1 — Table S1. Pairwise linkage disequilibrium analysis of 5 selected single nucleotide polymorphisms in As3MT among participants of University of Michigan Bladder Cancer Study. [file 1476-069X-11-43-S1.doc]

Supplementary Table 1. Pairwise linkage disequilibrium analysis of 5 selected single nucleotide polymorphisms in As3MT among participants of University of Michigan Bladder Cancer Study.

|  |  | rs1046778 | rs3740400 | rs11191439 | rs11191438 | rs7085104 |
| --- | --- | --- | --- | --- | --- | --- |
|  | r^2^ |  |  |  |  |  |
| rs1046778 |  | -- | 0.59 | 0.23 | 0.80 | 0.56 |
| rs3740400 |  |  | -- | 0.44 | 0.85 | 0.95 |
| rs1191439 |  |  |  | -- | 0.37 | 0.46 |
| rs1191438 |  |  |  |  | -- | 0.83 |
| rs7085104 |  |  |  |  |  | -- |
